# Complete Genome Sequence of Blautia producta JCM 1471^T^

**DOI:** 10.1128/MRA.00141-20

**Published:** 2020-04-23

**Authors:** Dieter M. Tourlousse, Mitsuo Sakamoto, Takamasa Miura, Koji Narita, Akiko Ohashi, Yoshihito Uchino, Atsushi Yamazoe, Keishi Kameyama, Jun Terauchi, Moriya Ohkuma, Hiroko Kawasaki, Yuji Sekiguchi

**Affiliations:** a Biomedical Research Institute, National Institute of Advanced Industrial Science and Technology (AIST), Tsukuba, Ibaraki, Japan; b Microbe Division/Japan Collection of Microorganisms, RIKEN BioResource Research Center, Tsukuba, Ibaraki, Japan; c Biological Resource Center, National Institute of Technology and Evaluation (NITE), Kisarazu, Chiba, Japan; d Japan Microbiome Consortium (JMBC), Osaka, Osaka, Japan; University of Maryland School of Medicine

## Abstract

We report a complete genome sequence of Blautia producta JCM 1471^T^. The genome consists of a single circular chromosome of 6,197,116 bp with a G+C content of 45.7%. The genome was annotated as containing 5 complete sets of rRNA genes, 70 tRNA genes, and 5,516 protein-coding sequences.

## ANNOUNCEMENT

The genus *Blautia* comprises some of the most abundant bacteria in the human gastrointestinal tract (1). *Blautia* spp. are thought to be important players in human health and disease based on the correlation between *Blautia* abundance and various health conditions, including diminished abundance in the elderly (2) and in patients with colorectal cancer (3). An abundance of *Blautia* was also associated with risk of death from acute graft-versus-host disease (4) and visceral fat accumulation (5). Within the genus, Blautia producta was identified as a key species responsible for restoration of colonization resistance against vancomycin-resistant enterococci (6), which was attributed to secretion of a lantibiotic (7). Here, we used Illumina and Oxford Nanopore Technologies (ONT) sequencing to generate a complete genome sequence of *B. producta* JCM 1471^T^, the authentic type strain of *B. producta*, formerly known as Ruminococcus productus (8).

Cells were obtained from the Japan Collection of Microorganisms and cultured under an N_2_ atmosphere in modified GAM broth with 1% glucose; DNA was purified using the EZ1 DNA tissue kit (Qiagen). Short-read Illumina libraries were constructed with the TruSeq Nano DNA kit and sequenced on a MiSeq instrument using V2 chemistry (2 × 251-bp reads) at a coverage of ∼100×. For ONT sequencing, libraries were prepared with the ligation sequencing kit (SQK-LSK109) and native barcoding expansion pack (EXP-NBD104). Sequencing was run on an R9.4.1 flow cell (FLO-MIN106) using the MinION device. Default parameters were used for all software for read processing and assembly unless indicated otherwise. Illumina sequencing reads were processed with Trimmomatic v0.38 (9), yielding 2,676,800 reads for assembly. For ONT sequencing, bases were called with Guppy v3.1.5 in high-accuracy mode, along with library demultiplexing and trimming of barcodes. After additional trimming of remaining barcodes with Porechop v0.2.4 (https://github.com/rrwick/Porechop) and removal of reads with a length of <1,000 bp or a quality score of <9 with NanoFilt v2.5.0 (10), Filtlong v0.2.0 (https://github.com/rrwick/Filtlong) was employed to eliminate the poorest 10% of read bases using the Illumina reads as references. This resulted in a total of 61,232 ONT reads with an *N*_50_ value of 7,955 bp and coverage of ∼70×. Hybrid assembly of the Illumina and ONT reads was performed with Unicycler v0.4.7 (11) and run in bold mode using a long-read assembly generated using Flye v2.5 (12). The NCBI Prokaryotic Genome Annotation Pipeline v4.11 (13) was used for genome annotation.

The genome assembly of *B. producta* JCM 1471^T^ consists of a single circular chromosome with a length of 6,197,116 bp and a G+C content of 45.7%. Annotation identified 5 complete sets of rRNA genes, along with 70 tRNA genes and 5,516 protein-coding sequences. The availability of a complete genome of *B. producta* JCM 1471^T^ will aid in advancing our understanding of the role of *B. producta* in the human gut by providing insights into its genomically encoded functional gene repertoire.

### Data availability.

The genome sequence described here has been deposited in DDBJ/EMBL/GenBank under the accession number CP048626. The raw Illumina and ONT sequencing reads are available in the Sequence Read Archive (SRA) under the accession numbers SRR10968453 and SRR10968454, respectively.
